# Discovery of novel peptides targeting pro-atherogenic endothelium in disturbed flow regions -Targeted siRNA delivery to pro-atherogenic endothelium *in vivo*

**DOI:** 10.1038/srep25636

**Published:** 2016-05-12

**Authors:** Jihwa Chung, Hyunbo Shim, Kwanchang Kim, Duhwan Lee, Won Jong Kim, Dong Hoon Kang, Sang Won Kang, Hanjoong Jo, Kihwan Kwon

**Affiliations:** 1 Medical Research Institute, School of Medicine, Ewha Womans University, Seoul,158-710, Republic of Korea; 2 Department of Internal Medicine, Cardiology Division, School of Medicine, Ewha Womans University, Seoul, 158-710, Republic of Korea; 3 Departments of Bioinspired Science and Life Science, Ewha Womans University, 11-1 Daehyun-dong, Seodaemoon-gu, Seoul, 120-750, Republic of Korea; 4 Department of Thoracic surgery, School of Medicine, Ewha Womans University, Seoul, 158-710, Republic of Korea; 5 Center for Self-assembly and Complexity, Institute for Basic Science (IBS), and Department of Chemistry, Pohang University of Science and Technology (POSTECH), Pohang 37673, Republic of Korea; 6 Department of Life Science, College of Natural Science, Ewha Womans University, 11-1 Daehyun-dong, Seodaemoon-gu, Seoul, 120-750, Republic of Korea; 7 Department of Biomedical Engineering, Georgia Institute of Technology and Emory University, Atlanta, Georgia, USA

## Abstract

Atherosclerosis occurs preferentially in arterial regions exposed to disturbed blood flow. Targeting these pro-atherogenic regions is a potential anti-atherogenic therapeutic approach, but it has been extremely challenging. Here, using *in vivo* phage display approach and the partial carotid ligation model of flow-induced atherosclerosis in mouse, we identified novel peptides that specifically bind to endothelial cells (ECs) exposed to disturbed flow condition in pro-atherogenic regions. Two peptides, CLIRRTSIC and CPRRSHPIC, selectively bound to arterial ECs exposed to disturbed flow not only in the partially ligated carotids but also in the lesser curvature and branching point of the aortic arch in mice as well as human pulmonary artery branches. Peptides were conjugated to branched polyethylenimine-polyethylene glycol polymer to generate polyplexes carrying siRNA targeting intercellular adhesion molecule-1 (siICAM-1). In mouse model, CLIRRTSIC polyplexes carrying si-ICAM-1 specifically bound to endothelium in disturbed flow regions, reducing endothelial ICAM-1 expression. Mass spectrometry analysis revealed that non-muscle myosin heavy chain II A (NMHC IIA) is a protein targeted by CLIRRTSIC peptide. Further studies showed that shear stress regulates NMHC IIA expression and localization in ECs. The CLIRRTSIC is a novel peptide that could be used for targeted delivery of therapeutics such as siRNAs to pro-atherogenic endothelium.

Atherosclerosis is a chronic immuno-inflammatory disease that preferentially occurs in disturbed flow regions where endothelial cells (ECs) are inflamed and dysfunctional^1^,^2^. Vascular ECs, which form the innermost layer of blood vessels, are exposed to fluid shear stress that modulates endothelial function and vascular pathophysiology^3^. It is well-known that expression of athero-protective genes is up-regulated by stable flow associated with physiologically high magnitude and unidirectional laminar shear stress (LSS)^4^; whereas pro-atherogenic genes are up-regulated by disturbed flow that are characterized by low and oscillatory shear stress (OSS)^5^,^6^. Clinically, atherosclerotic lesions develop predominantly in branched or curved regions of the associated with disturbed flow. Further, it was directly demonstrated that disturbed blood flow indeed induces atherosclerosis in hyperlipidemic ApoE^−/−^ mice^7^,^8^. Disturbed flow caused by partially ligating 3 of the 4 downstream branches of the left carotid artery (LCA), known as the partial carotid ligation model, rapidly induces robust atherosclerosis in the LCA, while the unligated right carotid artery (RCA) in the same animal remains plaque free^7^. This model further demonstrated that endothelial inflammation and dysfunction, which occur within one week following the partial ligation surgery, are critical events leading to atherosclerosis development^7^,^9^. These finding are consistent with the well-known importance of endothelial inflammation and endothelial dysfunction as critical events in the initiation and progression of human atherosclerosis^2^.

In the clinical setting, atherosclerosis is diagnosed using various imaging modalities, such as X-ray angiography, CT and MR angiography, and intravascular ultrasound. These imaging techniques measure luminal diameter, wall thickness, and plaque volume^10^,^11^,^12^,^13^. Recent efforts have focused on identifying a new atherosclerotic plaque-specific antigen, receptor, or other locally expressed biomarkers for more effective diagnosis and therapy in atherosclerosis. Moreover, effective and selective multifunctional nanocarriers have been discovered to deliver anti-atherogenic therapeutics to pro-atherogenic regions in atherosclerosis^14^,^15^,^16^,^17^,^18^,^19^,^20^,^21^. However, several challenges remain to be addressed in using selective nanocarriers as anti-atherogenic therapeutics, as systemic delivery of anti-atherogenic therapeutics can cause off-target effects not only in intended target cells but also in other tissues and cells.

The novelty of this paper is 1) discovery of new peptides expressed strongly in ECs exposed to pro-atherogenic flow conditions and 2) the successful use of these peptides as a targeted delivery of siRNAs to the ECs in the pro-atherogenic regions. Delivery of siRNAs *in vivo*, especially to dysfunctional ECs, continues to be a major obstacle in developing anti-atherogenic therapy.

Phage display provides a number of random peptides, which can be used as peptide ligands for targeting specific tissues and cells^22^,^23^,^24^. Peptides have several advantages as targeting molecules, including their small size, which provides better tissue penetration, higher biological activity, increased stability, and a reduced likelihood of unintended interaction with the immune system^25^. *In vivo* phage display is a powerful strategy for directly identifying peptides or proteins that target the vasculature of normal or diseased tissues in living animals^26^,^27^.

Here, we carried out *in vivo* phage display to identify novel peptides that bind specifically to pro-atherogenic arterial ECs in disturbed flow regions in mice. We identified several candidate peptides and demonstrated their selective and effective binding to pro-atherogenic ECs *in vivo* an *in vitro*. We further show that one of the peptides, CLIRRTSIC can be used to deliver siRNA to pro-atherogenic endothelium and effectively knockdown its target gene both *in vitro* and *in vivo*. And non-muscle myosin heavy chain IIA (NMHC IIA) is a protein targeted by CLIRRTSIC peptide in pro-atherogenic ECs exposed to disturbed flow.

## Results

### Selection of peptides specific to pro-atherogenic endothelium under disturbed flow

**Figure 1:**
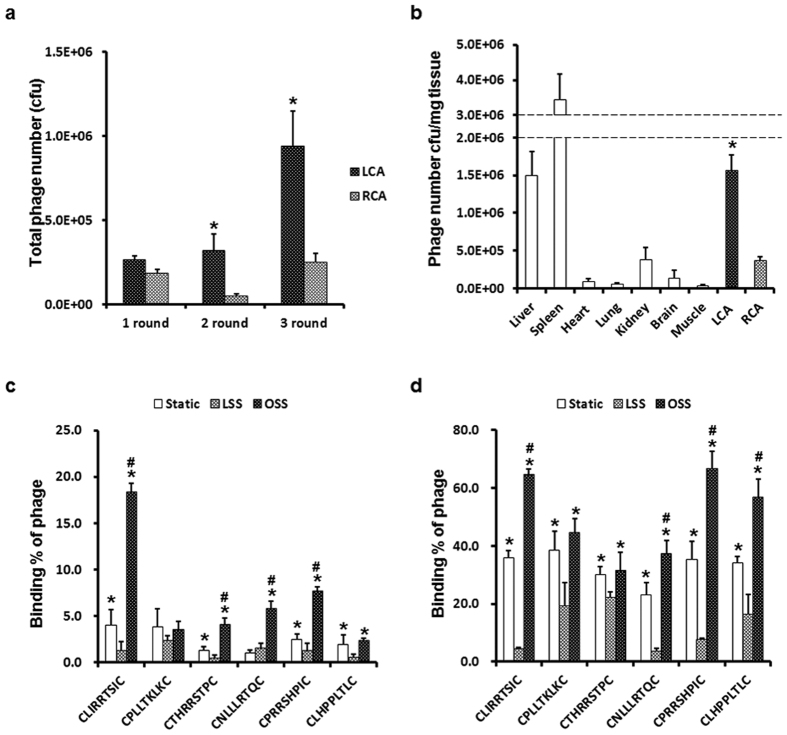
Selection and *in vitro* validation of specific phages for pro-atherogenic endothelium under disturbed flow. At 3 days post-partial ligation surgery on the left carotid ligation (LCA), the C57BL/6 mice (n = 3) were injected with a CX_7_C peptide library (1 × 10^11^ pfu) via the tail-vein. Ten minutes after the injection, the mice were perfused with saline and phages were recovered from various organs, including the carotid arteries. (**a**) Enrichment of phages in each screening round. Phages from the ligated LCA were recovered, amplified and re-injected in two consecutive rounds. Bars represent the titers of phages bound to the ligated LCA or non-ligated RCA in each selection round. Shown are means ± SEM of three independent experiments. *Significant difference (LCA vs. RCA, p < 0.05). (**b**) *In vivo* distribution of selected phages in mice. Organs were removed, homogenized and the number of phages in the tissues was determined at third rounds *in vivo* panning. At 3 days after surgery, the C57BL/6 mice (n = 3) were injected with eluted phages (1 × 10^11^ pfu) in second round. Ten minutes after the injection, the mice were perfused with saline and phages bound to organs including carotid arteries were collected. Bound phages were counted in various organs. Bars represent the titers of phages from the third round of amplification in various tissues. *Significant difference compared with other organs, with the exception of the liver and spleen (p < 0.05). (**c**,**d**) *In vitro* validation of individually selected phage clones in ECs. iMAECs (**c**) or HUVECs (**d**) were exposed to flow or static conditions for 48 h, and cells were incubated with each selected peptides (SPs)-displaying phage individually for 1 h. Cells were washed and bound phages were stained using an anti-M13 bacteriophage antibody. Bars represent the percent of phage positive ECs and are presented as means ± SEM of three independent experiments. *Significant difference compared with LSS conditions (p < 0.05). ^#^Significant difference compared with static conditions (p < 0.05).

To identify novel peptide targets that specifically bind to pro-atherogenic ECs in disturbed flow regions, we used the partial carotid ligation model of atherosclerosis that we developed^7^, which develops disturbed flow rapidly inducing endothelial dysfunction and atherosclerosis (within 2 weeks) in the entire length of left carotid artery (LCA) while the contralateral non-ligated right carotid artery (RCA) in the same mouse exposed to normal laminar flow remains plaque free. Three days after the partial carotid ligation, mice were injected with the Ph.D-C7C phage (1 × 10^11^ pfu) via tail-vein injection. Ten minutes later, LCAs were isolated and bound phages were flushed out, amplified in *E. coli*, and reinjected to the partially ligated mice. After three rounds of *in vivo* phage display, phage DNA of all colonies from the LCA and RCA were sequenced and selected for further analysis. Figure 1 shows the enrichment profile obtained in three rounds of selection. The total numbers of phages recovered from the ligated LCA markedly increased in the second and third rounds of selection, whereas the numbers of phages recovered from non-ligated RCA remained largely unchanged (Fig. 1a). We confirmed that the phages recovered from the ligated LCA targeted mainly the ligated LCA, except for tissues involved in the reticuloendothelial system (liver and spleen) and kidney after three rounds of selection (Fig. 1b). Through the sequencing analysis of phage DNAs, we identified six of selected peptides (SPs) that bound selectively to the ligated LCA (Table 1).

### *In vitro* validation of SPs

To determine whether six of SPs have specificity for pro-atherogenic ECs exposed to disturbed flow, the binding of homogeneous SPs-displaying phages was compared between ECs cultured under laminar shear stress (LSS) or oscillatory shear stress (OSS) conditions. We confirmed the effects of fluid shear stress first in immortalized mouse aortic endothelial cells (iMAECs) and also in human umbilical vascular endothelial cells (HUVECs) to examine whether the SPs can be used in both mouse and human tissues. LSS conditions induced morphological changes in ECs, causing elongation and alignment of their long axes in the direction of flow, but the OSS conditions resulted in irregular alignment (Supplementary Fig. 1a,c). We have previously shown the effects of shear stress on the expression of athero-protective and pro-atherogenic genes in ECs^28^. In this study, as expected, LSS increased the expression levels of athero-protective genes such as eNOS and KLF2, whereas OSS increased the expression levels of pro-atherogenic genes such as VCAM-1, ICAM-1 and MCP-1 (Supplementary Fig. 1b,d). iMAECs and HUVECs were cultured under LSS or OSS conditions for 48 h, and phage-binding assays were performed by immunofluorescence staining using an anti-M13 bacteriophage antibody without permeabilization. In iMAECs, CLIRRTSIC-, CTHRRSTPC-, CNLLLRTQC- and CPRRSHPIC-displaying phages showed significantly greater binding in cells exposed to OSS than LSS (Fig. 1c). On the other hand, CLIRRTSIC-, CNLLLRTQC-, CLHPPLTLC- and CPRRSHPIC-displaying phages bound significantly more to HUVECs exposed to OSS (Fig. 1d). Especially, CLIRRTSIC- and CPRRSHPIC-displaying phages showed most significant binding to both iMAECs and HUVECs exposed to OSS. These results indicate that these SPs-displaying phages bind specifically to ECs exposed to OSS, which supports the *in vivo* finding mimicking ECs exposed to disturbed flow conditions.

### *In vivo* validation of SPs

**Figure 2:**
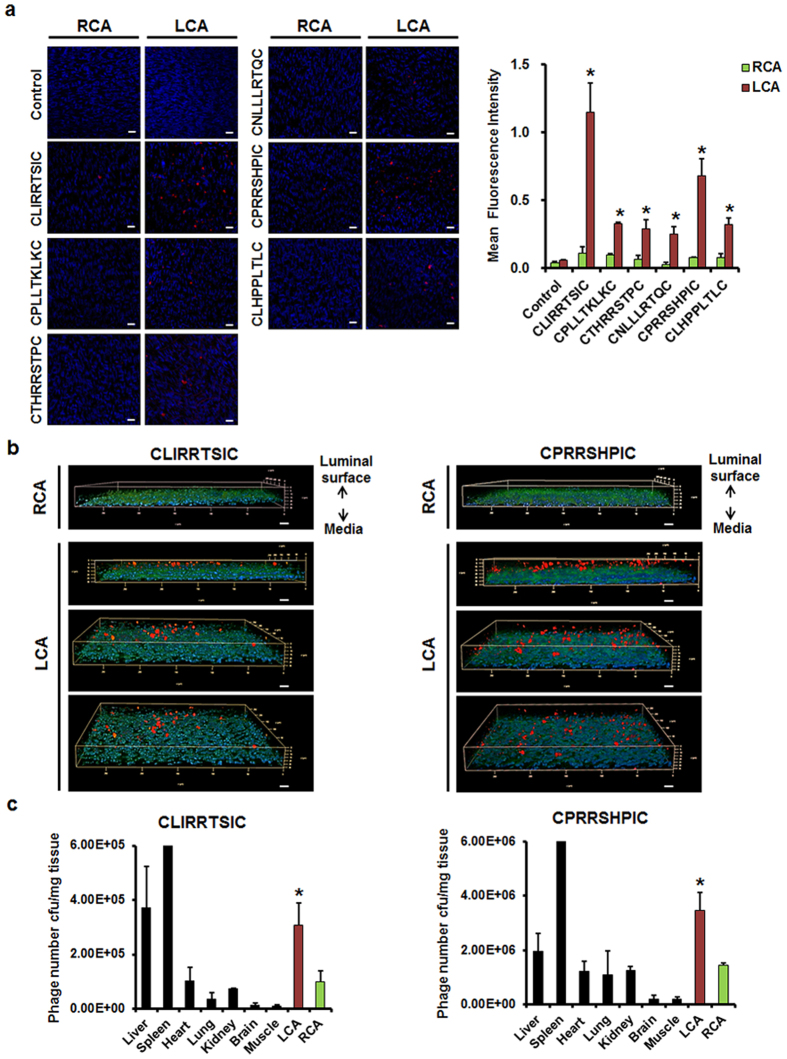
*In vivo* binding of SPs-displaying phages to the mouse carotid artery endothelium. (**a**) Validation of individual selected phage clones in the endothelium of mice. The control phages or six SPs-displaying phages (2 × 10^11^ pfu) were injected intravenously into C57BL/6 mice (n = 3) at 3 days after LCA ligation, and allowed to circulate for 10 min. Both carotid arteries were removed and the level of endothelium-bound phage was compared by *en face* staining. Carotid arteries were fixed and bound phages were stained using an anti-M13 bacteriophage antibody; nuclei were stained with DAPI (red: phage, blue: nuclei) (magnification, ×400; scale bars, 10 μm). Representative images are shown. Relative mean fluorescence intensity calculated using Image J for phage binding (red). *Significant difference (RCA vs. LCA, p < 0.05). (**b**) Localization of the phages attached on the endothelium. Phages attached to the luminal surface of the endothelium were verified by confocal microscopy z-stack imaging (red: phage, blue: nuclei, green: auto fluorescent of elastic lamina) (magnification, ×400; scale bars, 10 μm). Representative images are shown. (**c**) Bio-distribution of SPs-displaying phages in various organs including carotid arteries were quantified by titrating phage plaque numbers in the tissues obtained from the mice 10 min after the injection of two phages. Bars represent the titers of phages from the third round of amplification in various tissues and are presented as means ± SEM of three independent experiments. *Significant difference compared with other organs, with the exception of the liver and spleen (p < 0.05).

The endothelium of a partially ligated LCA was exposed to disturbed flow, which resulted in increased expression of VCAM-1 and ICAM-1 and atheroma formation within 2 weeks (Supplementary Fig. 2). These results are consistent with a previous study that used this animal model^7^,^20^. *In vivo* binding of SPs-displaying phages to pro-atherogenic endothelium exposed to disturbed flow in both carotid arteries and the aorta was compared by *en face* staining. The random phage library pool used as a control exhibited minimal binding to the cells. In carotid arteries, CLIRRTSIC- and CPRRSHPIC-displaying phages bound specifically to the ligated LCA, while binding to the non-ligated RCA was minimal (Fig. 2a). Additionally, phage-positive particles were localized on the luminal surface of endothelium in z-stack images (Fig. 2b). Furthermore, we examined the *in vivo* distribution of CLIRRTSIC- and CPRRSHPIC-displaying phages by titration of phage numbers in various organs of mice following intravenous injection. These two SPs-displaying phages targeted the pro-atherogenic endothelium of the ligated LCA, but no other organ except for liver and spleen (Fig. 2c).

**Figure 3:**
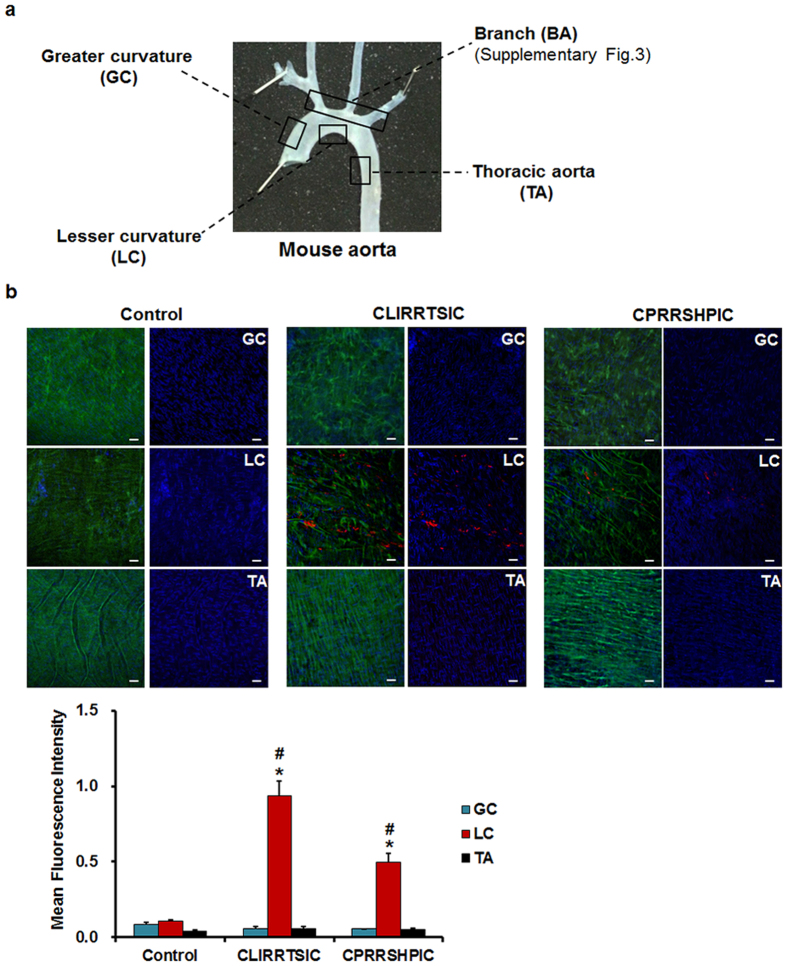
*In vivo* binding of SPs-displaying phages to the endothelium in various regions of the mouse aorta. The control phages, CLIRRTSIC- or CPRRSHPIC-displaying phages (2 × 10^11^ pfu) were injected intravenously into C57BL/6 mice (n = 3) at 3 days post-LCA partial ligation, and allowed to circulate for 3 h. (**a**) Mouse aorta. The aorta was divided into four regions according to the vessel wall shear stress distribution: branch (BA, disturbed flow), greater curvature (GC, normal laminar flow), lesser curvature (LC, disturbed flow) and thoracic aorta (TA, normal laminar flow). (**b**) The aorta was removed and the level of phage binding evaluated by *en face* staining. Aortic tissues were fixed and bound phages were stained using an anti-M13 bacteriophage antibody (red); nuclei were stained with DAPI (blue). Attached phages were observed in GC, LC and TA regions of mouse aorta by confocal microscopy (red: phage, blue: nuclei, green: elastic lamina of vessel) (magnification, ×400; scale bars, 10 μm). Representative images are shown. Relative mean fluorescence intensity calculated using Image J for phage binding (red). *^, #^Significant difference (*GC vs. LC; ^#^TA vs. LC, p < 0.05).

Since our partial carotid artery ligation model is a surgically induced model of disturbed flow, we then determined the binding of SPs-displaying phages to aortic tissues that are naturally exposed to disturbed flow, well-known pro-atherogenic regions *in vivo*, without surgery. The binding of phages to ECs in naturally disturbed flow regions that are also atherosclerosis-prone were compared to that of athero-resistant normal laminar flow regions: the branching points of arch vessels (BA exposed to disturbed flow), the greater curvature (GC exposed to normal laminar flow), lesser curvature (LC exposed to disturbed flow), and descending thoracic aorta (TA exposed to normal laminar flow) (Fig. 3a)^29^,^30^. We found that CLIRRTSIC- and CPRRSHPIC-displaying phages selectively bound to disturbed flow regions (LC) compared to the normal laminar flow regions (GC and TA), whereas the control phages did not (Fig. 3b). In addition, binding of these peptide-displaying phages was predominant in the disturbed flow regions near the orifice of BA than regions distant from orifice (Supplementary Fig. 3). Taken together, these results suggest that CLIRRTSIC- and CPRRSHPIC-displaying phages selectively bind to arterial endothelium exposed to disturbed flow induced by the partial carotid ligation surgery. More importantly, CLIRRTSIC- and CPRRSHPIC-displaying phages also bound to ECs in the curved and branching regions which are inherently and chronically exposed to disturbed flow regions and well-known pro-atherosclerotic regions with endothelial dysfunction, suggesting the potential importance of these peptides as specific targets spatially targeted delivery of therapeutics such as siRNAs to the pro-atherogenic ECs.

### Validation of SPs in human tissues

**Figure 4:**
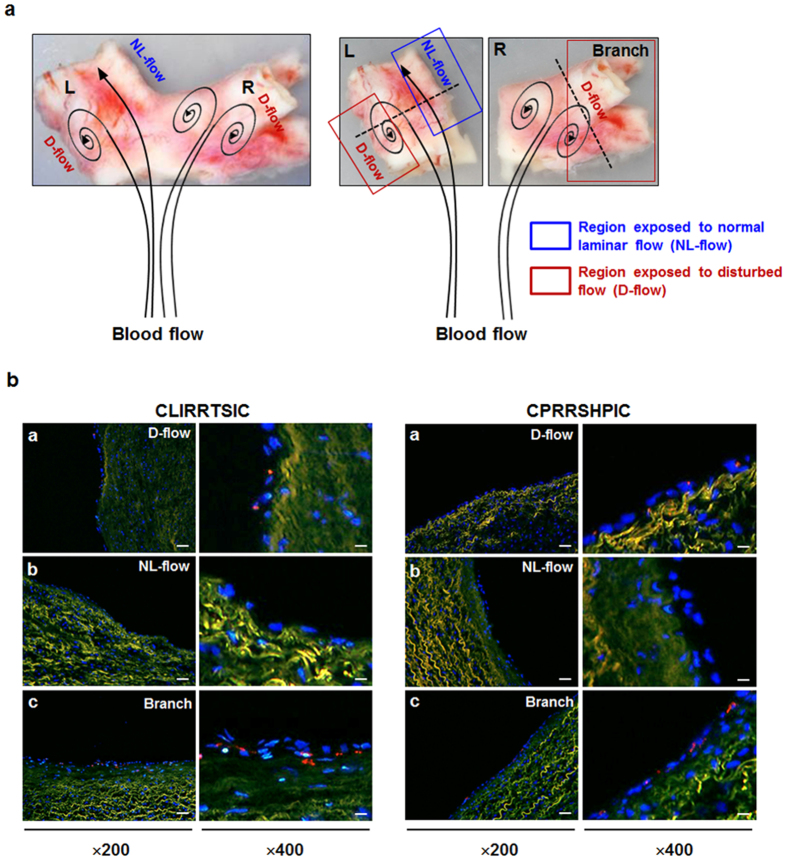
Binding of SPs-displaying phages in human tissue. Frozen sections of human tissues were incubated with 2 × 10^11^ pfu of CLIRRTSIC- or CPRRSHPIC-displaying phages for 3 h. (**a**) Appearance of an isolated human pulmonary artery. Human pulmonary artery was divided into two parts: the straight left (L) and right (R) parts with branch. The black arrow diagram appears the direction of blood flow. (**b**) Immunofluorescence staining for detection of phage binding in human tissue. Phage binding was compared in three regions with different wall shear stresses: the outer side with disturbed flow and inner side with normal laminar flow of the bifurcation site in left part (L) of specimen, and the branching regions with disturbed flow (Branch) in right part (R) of specimen using confocal microscopy (red: phage, blue: nuclei, green: elastic lamina of vessel) (magnification, ×200; scale bars, 20 μm, ×400; scale bars, 10 μm). Representative images are shown.

We next examined whether CLIRRTSIC- or CPRRSHPIC-displaying phages could bind to the endothelium in human pulmonary arterial branch regions that is expected to be exposed to disturbed flow (Fig. 4a). Both CLIRRTSIC- and CPRRSHPIC-displaying phages selectively attached to the endothelium in the lateral wall of the carina and branching points of the human pulmonary artery (Fig. 4b), while there were no detectable in regions exposed to normal laminar flow. These results indicate that CLIRRTSIC- and CPRRSHPIC-displaying phages can indeed bind to arterial ECs in disturbed flow regions in not only mouse arteries but also in human arteries, extending its potential application in human studies.

### siRNA delivery into the pro-atherogenic endothelium using SPs

Targeted delivery of siRNAs to pro-atherogenic endothelium *in vivo* is a major challenge currently, and if it can be overcome, it may provide an effective way to treat atherosclerosis in a gene-specific manner with minimized side effects. To this end, we tested whether our peptides identified here could be used as a novel targeted vehicle for siRNA delivery in mice. First, we examined whether synthetic, biotinylated SPs could selectively bind to ECs in disturbed flow regions compared to that of normal laminar flow *in vitro* and *in vivo*. For detection of biotinylated peptide binding, we used streptavidin-conjugated fluorephore quantum dots. To verify whether two peptides bind to surface of ECs, we performed immunofluorescence staining without permeabilization. Fluorescence microscopy studies showed that binding of the CLIRRTSIC and CPRRSHPIC peptides to OSS-cultured ECs was significantly higher than to LSS-cultured ECs, while binding of the CLNQQTAIC control peptide was minimal (Supplementary Fig. 4). These results suggest that synthetic CLIRRTSIC and CPRRSHPIC peptides are also specific for pro-atherogenic ECs exposed to disturbed flow, as are SPs-displaying phages.

Previously, we reported on an efficient gene delivery system (BPEI-SS-PEG-peptide) composed of bioreducible branched polyethyleneimine (BPEI-SS), polyethylene glycol (PEG) and targeting peptide with high transfection efficiency and targeting ability to tumor and brain tissues. BPEI-SS-PEG-peptides exist as stable polyplexes during circulation, but once they enter a cell, they are reduced and degraded by glutathione in intracellular compartments and release their cargo such as siRNA efficiently^31^,^32^.

To evaluate the efficacy of SPs to target pro-atherogenic ECs exposed to disturbed flow specifically, we determined siRNA internalization in ECs exposed to disturbed flow by using a SPs-conjugated BPEI-SS-PEG polymer. Peptides-conjugated polymers were synthesized by chemical coupling of the polymer and peptides (control CLNQQTAIC peptide, and SPs such as CLIRRTSIC and CPRRSHPIC) described in our previous study^32^. During thiolation, buffering capacity related with endosomal escape ability of entire polymer was kept owing to primary amine and secondary amine in BPEI was transformed to higher order amine. Degree of thiol, PEG and peptide was analyzed by ^1^H NMR (Supplementary Fig. 5 and Supplementary Table S1).

**Figure 5:**
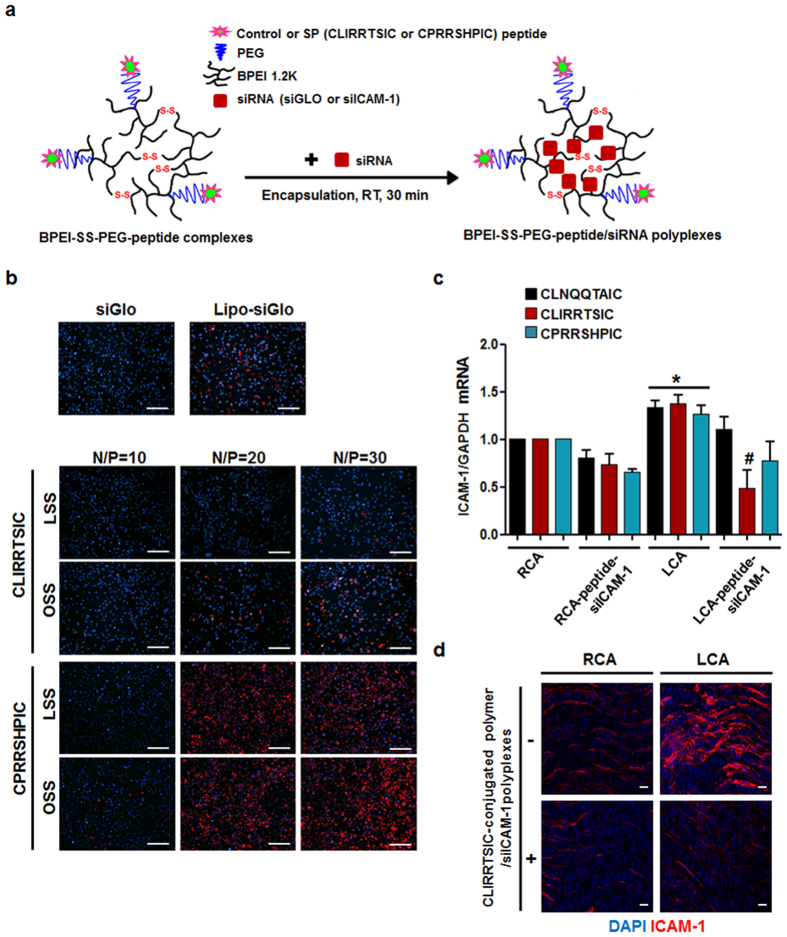
*In vitro* and *in vivo* delivery of siRNA to pro-atherogenic ECs using SPs-conjugated polymers. (**a**) Schematic diagram of BPEI-SS-PEG-SPs/siRNA polyplexes preparation. The SPs-conjugated polymers with fluorophore-labeled siRNA or anti-intercellular adhesion molecule-1 siRNA (siICAM-1) were prepared by mixing the components for 30 min at room temperature. (**b**) *In vitro* delivery of siRNA using peptides-conjugated polymers. HUVECs were exposed to static or flow conditions for 48 h. Cells were incubated with CLIRRTSIC- or CPRRSHPIC-conjugated polymer/siGLO polyplexes for 4 h at various N/P ratios. After 24 h, internalization of siGLO (red) was detected by immunofluorescence microscopy. Nuclei were stained with DAPI (blue) (magnification, ×100; scale bars, 100 μm). Representative images from at least three experiments are shown. (**c,d**) *In vivo* delivery of siRNA using peptides-conjugated polymers. siICAM-1 was encapsulated using CLIRRTSIC-, CPRRSHPIC- or control peptide CLNQQTAIC-conjugated polymers. Each peptide-conjugated polymer/siICAM-1 polyplexes were injected intravenously into mice (n = 5) at 3 days after partial ligation of the carotid artery. Twenty-four hours (**c**) after injection, mice were sacrificed and endothelial enriched RNAs obtained from LCA and RCA. The ICAM-1 mRNA level was determined by qPCR. Shown are means ± SEM of three independent experiments. *^, #^Significant difference (*RCA vs. LCA; ^#^LCA vs. LCA-peptide-siICAM-1, p < 0.05). Fourty-eight hours (**d**) after injection with CLIRRTSIC-conjugated polymer/siICAM-1 polyplexes, the carotid arteries were isolated and the protein levels of ICAM-1 evaluated by *en face* staining. Carotid arteries were fixed and ICAM-1 was stained using an anti-ICAM-1 antibody (red); nuclei were stained with DAPI (blue). Expression of ICAM-1 was observed by confocal microscopy (magnification, ×400; scale bars, 10 μm). Representative images are shown.

The peptide-conjugated polymers encapsulated with siGLO-red fluorophore-labeled siRNA or siICAM-1 in an adequate N/P ratio (Fig. 5a). The peptide-conjugated polymer/siGLO polyplexes were incubated with ECs exposed to LSS or OSS, and the cellular internalization of siGLO was examined. As shown in Fig. 5b, siGLO internalization increased in ECs exposed to OSS compared to LSS conditions predominantly in the case of the CLIRRTSIC-conjugated polymer. Cellular internalization of siGLO was dependent on the N/P ratio, but non-specific cellular internalization under LSS conditions increased with increasing N/P ratio, particularly in the case of the CPRRSHPIC-conjugated polymer.

Next, to determine the efficacy of homing or gene delivery by the SPs to pro-atherogenic ECs in mice with a partially ligated carotid artery, the control peptide- or SPs-conjugated polymer/siICAM-1 polyplexes were prepared by incubation at room temperature. Physicochemical properties of polyplexes were analyzed by performing gel retardation assay (Supplementary Fig. 6c) and measurement of size and zeta potential (Supplementary Fig. 6a,b). The control peptide- or SPs-conjugated polymer completely retard the siRNA at N/P >5 by gel retardation assay. Size and zeta potential of polymers also showed that all polymers could form nano-sized (~200 nm) and slightly positively charged (~15 mV) polyplexes at N/P > 5 and these results were correlated with gel retardation assay. Through experiments, we confirmed that the control peptide- or SPs-conjugated polymer is suitable candidates for *in vitro* and *in vivo* siRNA delivery.

The SPs- or control peptide-conjugated polymer/siICAM-1 polyplexes were intravenously injected into LCA-ligated mice, and the levels of mRNA and protein expression in ICAM-1 in ECs of both carotid arteries were compared. In the case of the CLIRRTSIC peptide, ICAM-1 expression in ligated LCA was selectively reduced in both mRNA and protein levels compared to the non-ligated RCA. However, there was no difference between the ligated LCA and non-ligated RCA following injection with the control CLNQQTAIC and CPRRSHPIC peptide-conjugated polymer/siICAM-1 complexes (Fig. 5c,d). These results suggest the CLIRRTSIC peptide to be able to specifically target pro-atherogenic ECs exposed to disturbed flow for drug or gene delivery.

### The CLIRRTSIC peptide binds to NMHC IIA

**Figure 6:**
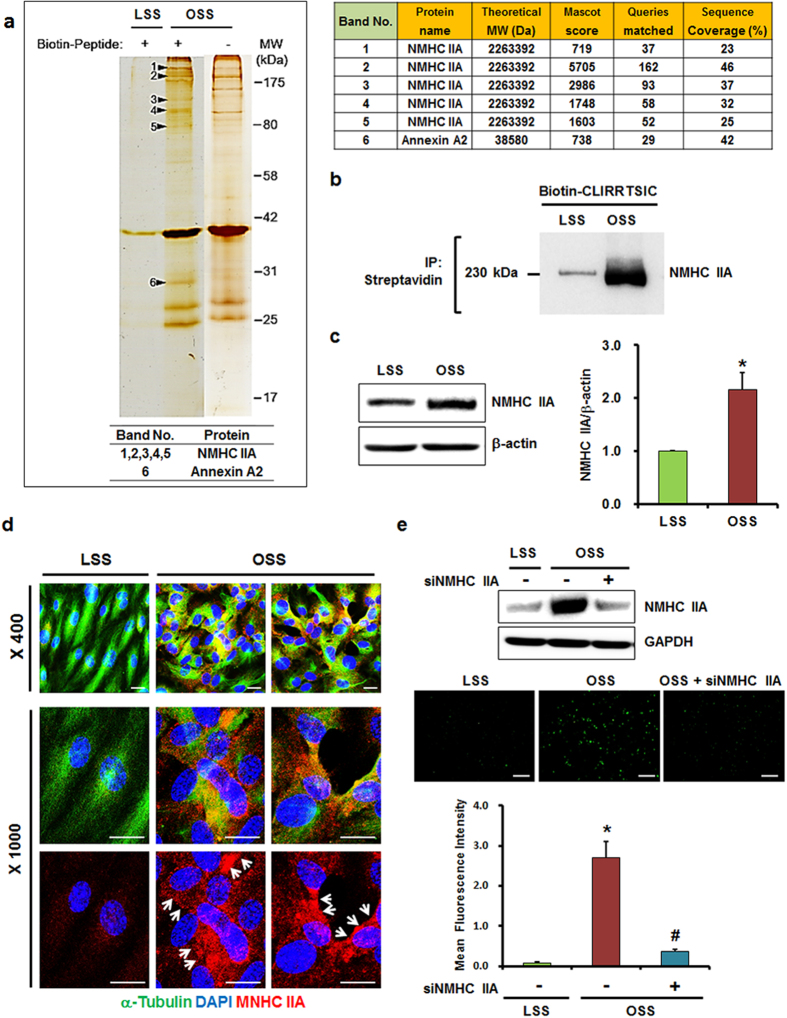
Identification of CLIRRTSIC peptide-binding target proteins. HUVECs were exposed to LSS or OSS for 48 h, and then incubated with or without biotinylated CLIRRTSIC peptide (20 μM) for 4 h. Cell lysates were incubated with streptavidin beads to pull down the biotinylated CLIRRTSIC peptide-binding proteins and were resolved by SDS-PAGE. (**a**) Proteomic analysis of CLIRRTSIC peptide-binding target protein. The gel was silver-stained and showed. Six proteins that were selected for sequencing by liquid chromatography-tandem mass spectrometry. MS/MS spectra of [M+H]^+^ ions of one of the peptides derived from the indicated protein is shown. (**b**) Binding of CLIRRTSIC peptide to NMHC IIA. HUVECs exposed to LSS or OSS for 48 h were incubated with biotinylated-CLIRRTSIC peptide for 4 h. The immunoprecipitates with streptavidin-beads were immunoblotted using an anti-NMHC IIA antibody. Shown is a representative of three independent experiments. (**c**,**d**) Expression of NMHC IIA in ECs. HUVECs exposed to LSS or OSS for 48 h. Cell lysates were immunoblotted and using an anti-NMHC IIA antibody (**c**). *Significant difference (LSS vs. OSS, p < 0.05). HUVECs exposed to LSS or OSS for 48 h were stained with anti-alpha tubulin and anti-NMHC IIA antibodies and observed using confocal microscopy (**d**). Shown are representative images of at least three experiments. Anti-alpha tubulin (green), anti-NMHC IIA antibody (red, arrows) and DAPI (blue) (magnification, ×400 or ×1000; scale bars, 10 μm). (**e**) HUVECs were transfected with siRNA of NMHC IIA (50 nM) for 48 h. After transfection, Cells were exposed to LSS or OSS for 48 h and then incubated with biotin-labeled CLIRRTSIC peptide (20 μM) for 4 h. Attached peptides were stained with streptavidin conjugated Qdot (green) (magnification, ×100; scale bars, 100 μm). Representative images are shown. Relative mean fluorescence intensity calculated using Image J for binding of CLIRRTSIC peptide (green). *^, #^Significant difference (*LSS vs. OSS; ^#^OSS vs. OSS + siNMHC IIA, p < 0.05).

To identify the target protein of the CLIRRTSIC peptide in OSS-exposed ECs, we examined the molecular composition of the CLIRRTSIC peptide-binding complex using a proteomics method that involved both immuno-affinity purification and mass spectrometry (M/S). As detailed in the Methods, confluent HUVECs were exposed to LSS or OSS for 48 h in the presence of biotinylated peptides, and the lysates were precipitated using streptavidin beads. Using the SDS-PAGE gel bands, liquid chromatography-tandem M/S analysis were carried out for six proteins. One protein we identified was NMHC IIA as a potential binding partner of SPs (Fig. 6a and Supplementary Fig. 7). To determine whether the biotinylated CLIRRTSIC peptide binds to NMHC IIA in HUVECs exposed to OSS, we performed immunoprecipitation using streptavidin beads and immunoblotting using an anti-NMHC IIA antibody. The density of the NMHC IIA band was markedly increased in OSS-exposed ECs. This suggests that the biotinylated peptide bound NMHC IIA in ECs exposed to OSS conditions (Fig. 6b). Next, we examined the expression and localization of NMHC IIA in ECs exposed to shear stress. As shown in Fig. 6c, the expression levels of NMHC IIA were higher in OSS-exposed ECs than in LSS-exposed ECs. As observed by immunofluorescence staining, NMHC IIA showed weak expression and was distributed evenly across the whole of LSS-exposed ECs. However, NMHC IIA showed increased expression and a propensity to be localized on the edge of plasma membrane in OSS-exposed ECs (Fig. 6d). Therefore, the expression of NMHC IIA increases and its cellular localization changes from the cytoplasm to the edge of plasma membrane in pro-atherogenic ECs exposed to OSS conditions. Next, to verify that NMHC IIA is a binding target for CLIRRTSIC, we performed examination for binding of CLIRRTSIC to ECs in which NMHC IIA has been knocked down with siRNA targeting NMHC IIA (siNMHC IIA). The increase in binding of CLIRRTSIC peptide to ECs exposed to OSS was disappeared in which NMHC IIA has been knocked down with siNMHC IIA (Fig. 6e). While this study cannot rule out the possibility that the peptide binds to the protein indirectly, the results taken collectively suggests that NMHC IIA may be a target of the CLIRRTSIC peptide, and may represent a novel target protein for pro-atherogenic ECs exposed to disturbed flow.

## Discussion

In the present study, using random phage libraries, we identified six novel peptide sequences that bind selectively to pro-atherogenic ECs exposed to disturbed flow. We confirmed that two of these novel peptides, CLIRRTSIC and CPRRSHPIC, bound specifically to arterial ECs in disturbed regions in not only mouse arteries but also in human arteries. Moreover, the CLIRRTSIC peptide-conjugated polymers/siICAM-1 polyplexes selectively homed to the endothelium of atheroprone regions with disturbed flow, delivering siICAM-1 and suppressing the levels of mRNA and protein expression in ICAM-1, which in ECs is increased by disturbed flow. These results suggest that the CLIRRTSIC peptide could be used for delivery of genes or therapeutic drugs specifically to the endothelium of atheroprone regions with disturbed flow.

Vascular ECs are directly exposed to changes in blood flow and shear stress. The shear stress of blood flow modulates the function of ECs by activating mechano-sensors, signaling pathways, and modulating the expression of genes and production of proteins. Disturbed blood flow with low OSS can cause endothelial dysfunction, and is a critical early event in atherogenesis^33^,^34^. Detection of these pro-atherogenic ECs in atheroprone regions is important for prevention, treatment and diagnosis of atherosclerosis. In this reason, recent studies have focused on identifying more effective and selective target molecules or nanocarriers to deliver anti-atherogenic therapeutics to pro-atherogenic regions in atherosclerosis. However, it has been extremely challenging.

Using MS/MS proteomic analysis, we found that the CLIRRTSIC peptide binds to the NMHC IIA protein. Furthermore, our results suggest that shear stress due to disturbed blood flow results in increased expression of NMHC IIA and a shift in its localization from the cytoplasm to the plasma membrane. In addition, we could confirm that the binding of CLIRRTSIC peptide to ECs exposed to OSS was disappeared in which NMHC IIA has been knocked down with siNMHC IIA. This suggests a possibility that NMHC IIA may be a novel protein involved in pro-atherogenic ECs in disturbed flow regions.

Non-muscle myosin II (NM II), which belongs to the myosin II subfamily, is an actin-binding protein and, similar to muscle myosin II, NM II molecules comprise three pairs of peptides: two heavy chains; two regulatory light chains, which regulate NMHC II activity; and two essential light chains that stabilize the heavy chain structure. Three genes in mammalian cells (myosin heavy chain: *Myh9*, *Myh10* and *Myh14*) encode NMHC II proteins (NMHC IIA, NMHC IIB and NMHC IIC, respectively)^35^. Previous studies reveled that NM II may also be activated directly by mechanical forces and accumulate at the point of mechanical perturbation, suggesting that NM II plays a central role in the cellular response to mechanical stimulation due to its ability to generate mechanical force^35^,^36^. Although NMHC IIA plays a basic role in processes that require cellular reshaping and movement, such as cell adhesion and cell migration^35^,^37^, few studies have evaluated its role in ECs and its effect on the function of the endothelium under shear stress. In this study, we report for the first time that NMHC IIA in ECs responds to changes in shear stress. Therefore, NMHC IIA has a role in endothelial dysfunction induced by disturbed flow and development of atherosclerosis from an early stage. Further studies are required to elucidate the function of NMHC IIA in pro-atherogenic ECs exposed to disturbed flow, which may increase our understanding of the pathophysiology of atherosclerosis induced by disturbed flow, and facilitate identification of new target molecules for novel therapies.

Many studies have emphasized the identification of target molecules specific to tissues or cell types as an essential component of drug delivery or development of new therapeutic agents. These molecules improve the efficacy of drug or gene delivery to target sites, and may minimize adverse effects on non-target regions^38^,^39^. In this study, we identified NMHC IIA to be a target protein of pro-atherogenic ECs exposed to disturbed blood flow and a biomarker of early atherosclerotic lesions.

In conclusion, our study demonstrated that *in vivo* phage display facilitates identification of peptides that bind selectively to pro-atherogenic ECs in disturbed flow regions. These peptides, CLIRRTSIC and CPRRSHPIC, preferentially bound to ECs exposed to disturbed flow *in vitro* and in mouse and human arterial tissues. The CLIRRTSIC peptide exhibited the greatest selectivity for pro-atherogenic ECs exposed to disturbed flow and may be suitable for delivery of genetic material, such as siRNA, into pro-atherogenic endothelium in atheroprone regions with disturbed flow *in vivo*. Moreover, the CLIRRTSIC peptide binds to NMHC IIA; therefore, this may be a new target protein for pro-atherogenic ECs in disturbed flow regions that are also atheroprone regions with endothelial dysfunction. Taken together, our findings suggest the CLIRRTSIC peptide and NMHC IIA to be useful for selective delivery of therapeutic drugs, genes and imaging probes to pro-atherogenic endothelium in atherosclerosis.

## Methods

### Cell culture and shear stress experiments

Immortalized mouse aortic endothelial cells (iMAECs)^40^ and human umbilical vein endothelial cells (HUVECs)^28^,^41^ were cultured as described in a previous study. Confluent iMAECs or HUVECs were exposed to fluid shear stress as indicated. Cells were exposed to flow in a cone-and-plate viscometer. We used a unidirectional steady flow (shear stress of 25 dyne/cm^2^) for laminar shear stress (LSS), and a bidirectional disturbed flow (shear stress of ± 5 dyne/cm^2^) for oscillatory shear stress (OSS), for 48 h^28^.

### Animal model of atherosclerosis induced by disturbed flow

All procedures performed on the animal models were approved by Ewha Womans University Animal Ethics Committee, and experiments were conducted in accordance with the approved guidelines. We generated a model of atherosclerosis induced by disturbed flow in mice by partial carotid artery ligation. Male C57BL/6 (Central Lab Animal, Korea) mice were ligated at 6 weeks of age. In this model, the endothelium of the partially ligated LCA was exposed to disturbed flow characterized by low and oscillating shear stress, endothelial dysfunction was induced in 1 week and atherosclerosis developed within 2 weeks. All mice were fed a chow diet and water *ad libitum*. Partial ligation of the LCA was carried out as described in a previous study^7^,^28^,^42^.

### *In vivo* phage display

We used the Ph.D-C7C phage display peptide library kit (New England Biolabs, USA), and screening using the phage display library was performed according to the manufacturer’s instructions. Briefly, 1 × 10^11^ plaque-forming units (pfu) of phages were injected intravenously into mice at 3 days after LCA ligation. After 10 minutes of circulation, the mice were anesthetized with zoletil and rompun, and pressure perfused with saline containing heparin (10 U/ml) via the left ventricle after severing the inferior vena cava to remove unbound phage. The LCA and the RCA were then isolated. The carotid lumen was rapidly flushed using a 29-gauge insulin syringe with 200 μL of phage elution buffer (0.2M Glycine-HCl, pH 2.2) for detachment of bound phages from endothelium and subsequently neutralized with 30 μL of 1 M Tris-HCl (pH 9.1) using a 29-gauge insulin syringe into a microfuge tube. Eluted phages from LCA were amplified in *Escherichia coli* ER2738 for reinjection in the next round. After three rounds of phage propagation, individual blue *E. coli* colonies that bound to LCA or RCA were picked and further amplified for isolation of phage DNA. Six peptide sequences were picked from the sequenced phage DNAs that selectively attached to the LCA compared to the RCA. For further binding studies, high-titer homogeneous populations of each selected peptides (SPs)-displaying phage were prepared.

### *In vitro* phage binding assay

For *in vitro* binding studies of SPs-displaying phages, iMAECs and HUVECs were exposed to LSS or OSS for 48 h. The cells were biopanned individually for 1 h with 6 × 10^10^ pfu of each SPs-displaying phage at 37 °C. Cells were fixed and blocked with 10% donkey animal serum in PBS. For detection of phages bound to surface of ECs, immunofluorescence staining was performed without permeabilization. Cells were incubated with an anti-M13 bacteriophage antibody (1:200, Abcam, USA) overnight at 4 °C. Anti-mouse IgG-R antibody (1:500, Santa Cruz Biotechnology, USA) was used as a secondary antibody. Phages bound to ECs were detected by Olympus BX51 (Olympus America). All images were analyzed with Motic Images Plus software (MIPlus 2.0) and the percent of phage positive ECs was quantified.

### *In vivo* phage binding assay

Three days after carotid artery ligation surgery, 2 × 10^11^ pfu of SPs-displaying phages were injected intravenously and allowed to circulate for 10 min. The carotid arteries and aorta were collected for *en face* staining. Isolated carotid arteries and aorta were fixed. After blocking with 10% donkey animal serum in PBS, tissues were incubated with an anti-M13 bacteriophage antibody (1:200, Abcam, USA) overnight at 4 °C. The tissues were then incubated with a secondary anti-mouse IgG-R antibody (1:500, Santa Cruz Biotechnology, USA) for 2 h at room temperature. Phages attached on the endothelium were detected by LSM Pascal confocal microscope (Carl Zeiss, Germany). For Z-stack imaging of phage localization on endothelium, tissues were observed on Leica TCS SP8 confocal laser scanning microscope (Leica, Germany). All images were analyzed with Leica software (LAS X) and final images were obtained by using Huygens software.

### Phage binding in human arterial tissue

With informed consent, human lobar pulmonary arteries were harvested from a patient who underwent a pulmonary lobectomy and immediately frozen at −80 °C. All procedures performed on human experiments were approved by the Research Ethics Committees of Ewha Womans University, and experiments were conducted in accordance with the approved guidelines. Frozen sections were incubated with 3 × 10^11^ pfu of SPs-displaying phages for 3 h at 37 °C and then washed to remove unbound phage. After fixation, frozen sections were blocked and incubated with an anti-M13 bacteriophage antibody (1:200, Abcam, USA) overnight at 4 °C. Anti-mouse IgG-R antibody (1:500, Santa Cruz Biotechnology, USA) was used as a secondary antibody. DAPI was used for nuclear counterstaining. After mounting, tissue slides were visualized by fluorescence microscopy.

### Synthesis of SPs-conjugated polymer (BPEI-SS-PEG)

Branched polyethylenimine (BPEI) 1.2K was purchased from Polyscience. Propylene sulfide and dimethyl sulfoxide (DMSO) were purchased from Sigma-Aldrich (St Louis, MO). Heterobifunctional polyethylene glycol, α-maleimide-ω-N-hydroxysuccinimide ester polyethylene glycol (MAL–PEG–NHS) was purchased from NOF Corporation (Tokyo, Japan). The control CLNQQTAIC peptide and SPs such as CLIRRTSIC and CPRRTSIC were synthesized by AnyGen (Jangsung, Korea). Thiol moieties were conjugated to the primary and secondary amine groups of BPEI1.2K using propylene sulfide as a thiolation reagent to produce thiolated BPEI (BPEI-SH). To synthesize disulfide cross-linked BPEI (BPEI-SS), BPEI-SH was dissolved in DMSO and the solution was stirred for 48 h at room temperature for crosslinking via oxidation of thiol groups. BPEI-SS was dialyzed against DI water for 48 h to remove unreacted BPEI-SH, and then lyophilized. Next, the peptides and NHS-PEG-MAL were dissolved in DMSO and stirred for 4 h at room temperature, followed by addition of BPEI-SS in DMSO. The reaction mixture was stirred for a further 48 h at room temperature to produce BPEI-SS-PEG-peptides. Final products were purified by dialyzing against DI water, and then lyophilized. The detailed method for synthesis of polymer and conjugation with peptide were described previously^31^,^32^.

### Preparation of peptides-conjugated polymer/siRNA polyplexes

For preparation of polyplexes, peptides-conjugated polymers with fluorophore-labeled siRNA (siGLO Red Transfection indicator; Dharmacon, USA) and siRNA against intercellular adhesion molecule-1 siRNA (siICAM1) were prepared by mixing the components at N/P ratios (the ratios of concentrations of total nitrogen atoms (N) of the polycation to the phosphate groups (P) of siRNA) of 10 to 30 for 30 min at room temperature, and then used for *in vitro* and *in vivo* delivery assays.

### Gel retardation assay in polyplexes

The polyplexes were prepared by adding the polymer solution to the siRNA solution and were adjusted according to the various N/P ratios. Polyplexes were loaded onto 13% polyacrylamide gel with 6× loading dye. Electrophoresis was carried out at a constant voltage of 100 V for 90 min in 1.0× TBE buffer (Tris-borate-EDTA). After electrophoresis, the gel was incubated in SYBR green solution. The bands were visualized under UV illuminator and analyzed using a Davinch Western Imaging System (Davinch-K, Younghwa Science, Korea).

### Size and zeta potential measurements in polyplexes

The polyplexes were prepared at a various N/P ratio from 1 to 30 by adding the polymer solution to the siRNA solution and diluted by DEPC water. The final siRNA solution was adjusted to 100 nM. The mixture was then incubated for 30 min at room temperature. The particle size of each samples were measured by particle size analyzer using Zetasizer Nano S90 (Malvern Instruments, Malvern, U.K.). And zeta potential of each sample was measured by using a Zetasize Nano Z (Malvern Instruments, Malvern, U.K.).

### *In vitro* delivery of siRNA using SPs-conjugated polymer

To analyze efficiency of selective siRNA delivery into ECs by SPs-conjugated polymers, we examined cellular internalization of the fluorophore-labeled siRNA (Dharmacon, USA) in ECs. HUVECs were exposed to LSS or OSS for 48 h. siGLO (50 nM), either free or complexed with SPs-conjugated polymer (N/P ratios of 10 to 30), was added to cells and incubated for 4 h. As a positive control for intracellular siGLO delivery, cells were transfected using Lipofectamine 2000 (Invitrogen, USA), as described by the manufacturer. After 24 h, intracellular siGLO was detected using fluorescence microscopy.

### *In vivo* delivery of SPs-conjugated polymer/siRNA polyplexes to pro-atherogenic endothelium

For the *in vivo* delivery study, the control CLNQQTAIC peptide- or SPs-conjugated polymer/siICAM−1 (30 μg, N/P = 20) polyplexes were intravenously injected via tail-vein at 3 days after LCA ligation. Total RNA was extracted from ECs of the ligated LCA or non-ligated RCA at 24 h after injection. The partially ligated LCA and non-ligated RCA were isolated and carefully cleaned of periadventitial fat as described previously^7^. The lumen of both carotid arteries was rapidly flushed with 200 μL of QIAzol lysis reagent (Qiagen, USA). The eluate was then used for endothelial enriched RNA isolation using a miRNeasy mini kit (Qiagen, USA) according to the manufacturer’s instructions. The expression levels of ICAM-1 mRNA in carotid artery endothelial enriched RNA preparation were determined by qRT-PCR (StepOne Real-time PCR system; Applied Biosystems, USA). To detect the expression of ICAM-1 protein, CLIRRTSIC-conjugated polymer/siICAM-1 (30 μg, N/P = 20) polyplexes were intravenously injected via tail-vein at 3 days after LCA ligation. Carotid arteries were collected at 48 h after injection for *en face* staining. Isolated carotid arteries and aorta were fixed. After blocking with 10% donkey animal serum in PBS, tissues were incubated with an anti-ICAM-1 antibody (1:50, SouthernBiotech, USA) overnight at 4 °C. The tissues were then incubated with a secondary Rhodamine Red-X (RRX) anti-rat IgG antibody (1:500, Jackson ImmunoResearch, USA) for 2 h at room temperature. Expression of ICAM-1 on the endothelium was detected by LSM Pascal confocal microscope (Carl Zeiss, Germany).

### Immunofluorescence staining for NMHC IIA

HUVECs were exposed to LSS or to OSS conditions for 48 h. After fixation, cells were blocked and incubated with alpha-tubulin (1:200, Sigma-Aldrich, USA) and anti-NMHC IIA antibody (1:50, Cell Signaling Technology, USA) overnight at 4 °C. Anti-mouse IgG-Alexa 488 (1:500, Molecular Probe, USA) and anti-rabbit IgG-R antibody (1:200, Santa Cruz Biotechnology, USA) was used as a secondary antibody. DAPI was used for nuclear counterstaining. After mounting, expression and localization of NMHC IIA was visualized by fluorescence microscopy.

### Mass spectrometry (M/S)

For sample preparation, HUVECs were cultured under LSS or OSS conditions for 48 h and then incubated for 4 h with 20 μM of biotin-labeled CLIRRTSIC peptide at 37 °C. Cell extracts were prepared using lysis buffer. For immunoprecipitation, cell extracts (1.6 mg protein) were pre-cleared with 20 μL of protein-A/G Sepharose 4 Fast Flow beads (Amersham Biosciences, Sweden) for 1 h. The resulting supernatant was incubated overnight with 3 μg of streptavidin beads with rotation, and then mixed with 20 μL of protein-A/G beads at 4 °C for an additional 3 h. Immunocomplexes were washed three times with 1 mL of extraction buffer. Proteins were then separated on a 10% SDS-PAGE gel and subsequently stained with silver nitrate. The silver-stained spots were subjected to in-gel trypsin digestion with the following minor modifications. Briefly, the gel spots were excised with a scalpel and destained by washing with 15 mM K_4_Fe(CN)_6_ and 50 mM sodium thiosulfate. The gel pieces were crushed, dehydrated by adding acetonitrile, rehydrated by adding 10–20 mL of 25 mM ammonium bicarbonate with 10 ng/mL of sequencing-grade trypsin (Promega, USA), and incubated for 15–17 h at 37 °C. The peptides in the supernatant were transferred to a clean tube and extracted twice by adding 50 mL of a solution containing 60% acetonitrile and 0.1% trifluoroacetic acid. The extracted solutions were pooled and evaporated to dryness in a SpeedVac vacuum centrifuge. Peptide sequencing was performed by tandem mass spectral (MS/MS) analysis using nano-flow reversed-phased HPLC/ESI/MS and a mass spectrometer (Q-TOF Ultima™ global, UK). Peptides were separated using a C18 reversed-phase 75-mm i.d. 6150 mm analytical column (3-mm particle size, Atlantis™ dC18, Waters, USA) with integrated electrospray ionization Silica-Tip™ (610 mm, New Objective). In detail, 5 mL of peptide mixtures were dissolved in buffer A (water/ACN/formic acid; 95:5:0.2, v/v), injected onto a column and eluted using a linear gradient of 5–80% buffer B (water/ACN/formic acid; 5:95:0.2, v/v) over 120 min. Samples were desalted on a line prior to separation using a trap column (i.d. 0.35650 mm, OPTI-PAKTM C18, Waters, USA) cartridge. Initially, the flow rate was set to 200 nL/min by a split/splitless inlet and the capillary voltage (3.0 keV) was applied to the HPLC mobile phase before spraying. Chromatography was performed online using the Q-TOF Ultima™ global instrument’s control software, MassLinx. The mass spectrometer was programmed to record scan cycles composed of one MS scan followed by MS/MS scans of the eight most abundant ions in each MS scan. MS parameters for efficient data acquisition were intensity (0.10) and number of components (3, 4) to be switched from an MS to MS/MS analysis. Following positive identification, all peptides identified by means of a database search (Mascot) were excluded from the next analysis run until full sequence coverage was obtained. Database analyses using the database search software Mascot (Global search engine), Proteinlynx 2.1 (Waters, USA) and MODi (Korea, http://modi.uos.ac.kr/modi/), provided almost full sequence coverage on selective exclusion monitoring. MS/MS spectra were matched against amino acid sequences in SwissProt. Precursor ion mass corrections and a fragment ion mass tolerance of 0.2 Da were used to indicate two missed cleavages.

### Statistical analyses

All data are expressed as means ± SEM of at least three independent experiments of the given number. Differences in quantitative variables were tested using a nonparametric Mann–Whitney U-test. Differences between groups were considered to be significant at *p* < 0.05.

## Additional Information

**How to cite this article**: Chung, J. *et al.* Discovery of novel peptides targeting pro-atherogenic endothelium in disturbed flow regions - Targeted siRNA delivery to pro-atherogenic endothelium *in vivo*. *Sci. Rep.* **6**, 25636; doi: 10.1038/srep25636 (2016).

## Supplementary Material

Supplementary Information

## Figures and Tables

**Table 1:** Alignment of phage-displayed peptide sequences selected from a ligated LCA or non-ligated RCA.

	LCA (disturbed)	RCA (Normal)		LCA (disturbed)	RCA (normal)
	(Frequency)/123	(Frequency)/107		(Frequency)/123	(Frequency)/107
CKMTRSTIC	29	22	CRTKLRKLC	1	
CKIMISISC	19	30	CHMQIMHRC	1	
CLIRRTSIC	8	2	CTRMMLRIC	1	
CPLLTKLKC	8	4	CTPRQTRMC	1	
CTHRRSTPC	7	5	CMLNITRRC	1	
CHIPSIHSC	8	8	CRKLPRISC	1	
CQMMLIRRC	4	4	CPLQPKSIC	1	
CNLLLRTQC	5	1	CPIRHRPIC	1	
CPRRSHPIC	4		CRTMRIRLC	1	
CLHPPLTLC	6	2	CMRQRRNRC		2
CKMNIRLSC	2		CPRRRLKRC		1
CILRKIRPC	4	4	CHQLPIRPC		1
CSRRPTSIC	1	1	CPIKTTITC		1
CLIRLILQC	1	2	CRRQTSTHC		1
CRMRKTLRC	1	1	CNPMTRLIC		2
CQPHHSIIC	1	1	CPIRHRPIC		1
CSTHIPSHC	1	1	CTLHKSRSC		1
CILRRRKRC	1	1	CTRIISNTC		1
CSIRIHRRC	1		CKLINTLKC		1
CSLRRHPIC	1		CQMTRSTIC		1
