# Supplementary material for: Discovery of novel peptides targeting pro-atherogenic endothelium in disturbed flow regions -Targeted siRNA delivery to pro-atherogenic endothelium in vivo
Source: Sci Rep. 2016 May 12;6:25636. doi: 10.1038/srep25636 (PMC4901192; doi:10.1038/srep25636)
Supplement: Supplementary Information [file srep25636-s1.pdf]

Discovery of novel peptides targeting pro-atherogenic endothelium in disturbed flow regions - Targeted siRNA delivery to pro-atherogenic endothelium *in vivo*

Jihwa Chung [1], Hyunbo Shim[3], Kwanchang Kim[4], Duhwan Lee[5], Won Jong Kim[5], Dong Hoon Kang[6], Sang Won Kang [6], Hanjoong Jo[7] and Kihwan Kwon[1,2 *]

[1]Medical Research Institute, School of Medicine, Ewha Womans University, Seoul,158-710, Republic of Korea.

[2]Department of Internal Medicine, Cardiology Division, School of Medicine, Ewha Womans University, Seoul, 158-710, Republic of Korea

[3]Departments of Bioinspired Science and Life Sciences, Ewha Womans University, 11-1 Daehyun-dong, Seodaemoon-gu, Seoul, 120-750, Republic of Korea

[4]Department of Thoracic surgery, School of Medicine, Ewha Womans University, Seoul, 158-710, Republic of Korea

[5]Department of Chemistry, Pohang University of Science and Technology, San 31, Hyoja-dong, Pohang, 790-784, Republic of Korea

[6]Department of Life Science, College of Natural Science, Ewha Womans University, 11-1 Daehyun-dong, Seodaemoon-gu, Seoul, 120-750, Republic of Korea

[7]Department of Biomedical Engineering, Georgia Institute of Technology and Emory University, Atlanta, Georgia, U.S.A

* Corresponding author. Department of Internal Medicine, Cardiology Division, School of Medicine, Ewha Womans University, Seoul, 158-710, Republic of Korea. Phone: 82-2-2650-2023. Fax: 82-2-2650-2567. E-mail:kankadin@ewha.ac.kr

Supplementary information

Supplementary Table S1. Characterization of BPEI-SS-PEG-peptides polymer.

	BPEI-SS-PEG-CLNQQTAIC	BPEI-SS-PEG-CLIRRTSIC	BPEI-SS-PEG-CPRRSHPIC
Thiol/BPEI 1.2k [a]		5.12	
PEG/BPEI 1.2K [b]	0.11	0.1	0.12
Peptide/BPEI 1.2k [c]	0.28	0.24	0.37

The conjugation ratio was calculated by analyzing ^{1}H NMR spectra of each polymer (BPEI-SS-PEG-CLNQQTAIC, BPEI-SS-PEG-CLIRRTSIC or BPEI-SS-PEG-CPRRSHPIC), respectively. [a] Molar ratio of thiol groups to BPEI 1.2k chain in polymers. [b] Molar ratio of PEG to BPEI 1.2k. [c] Molar ratio of peptide to BPEI 1.2k.

.

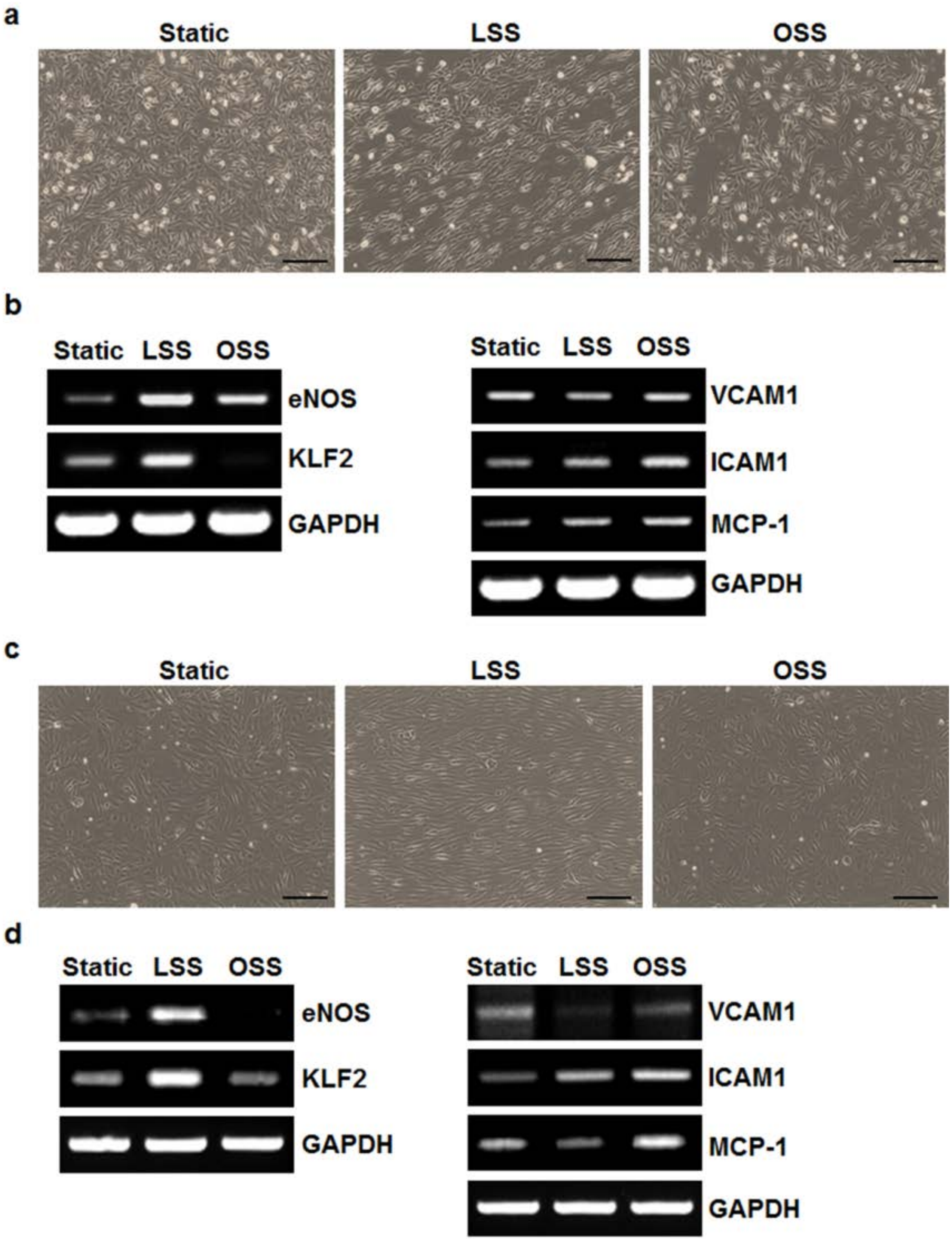

Supplementary Figure 1. Effects of fluid shear stress on ECs.

(a and c) Microscopic analysis of morphological changes in iMAECs (a) and HUVECs (c)

cultured under flow or static conditions for 24 h (magnification, ×100; scale bars, 100 μm). (b and d) Reverse transcription (RT)-PCR of anti-inflammatory (eNOS and KLF2) and pro-inflammatory (VCAM-1, ICAM-1 and MCP-1) genes involved in atherosclerosis. iMAECs (b) and HUVECs (d) were cultured under flow or static conditions for 6 h.

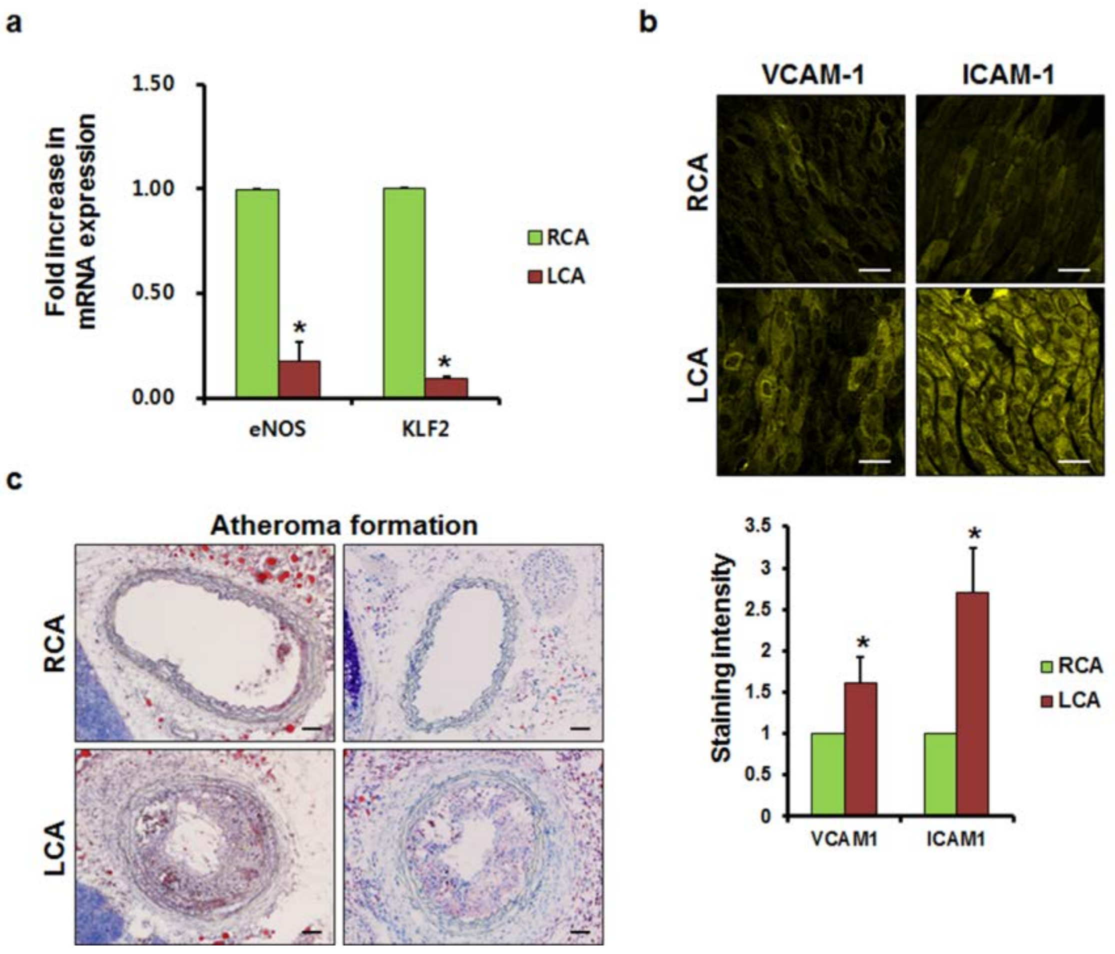

Supplementary Figure 2. Characteristics of endothelium under disturbed flow due to partial ligation of the carotid artery.

(a) Expression of anti-inflammatory genes (eNOS and KLF2) in carotid arteries. Carotid arteries of mice (n=3) were isolated and total RNA was extracted from ECs of ligated LCA or non-ligated RCA at 3 days after ligation. The eNOS and KLF2 mRNA levels in ECs of carotid arteries were determined by qRT-PCR. Bars represent the LCA/RCA ratio of intensity values as means ± SEM of three independent experiments. * Significant difference compared with RCA ($p<0.05$). (b) Expression of VCAM-1 and ICAM-1 in carotid arteries. The carotid arteries of C57BL/6 mice were removed at 3 days after LCA ligation for *en face* staining and immunostaining of VCAM-1 and

ICAM-1 (magnification, ×630; scale bars, 10 μm). Bars represent the LCA/RCA ratio of intensity values as means±SEM of three independent experiments. * Significant difference compared with RCA ($p<0.05$). (c) Atheroma formation by partial ligation of the carotid artery. ApoE KO mice were partially ligated and fed a high-fat diet for 2 weeks. Frozen sections of carotid arteries were stained with Oil red O (magnification, ×200; scale bars, 20 μm). Representative images of at least five experiments are shown.

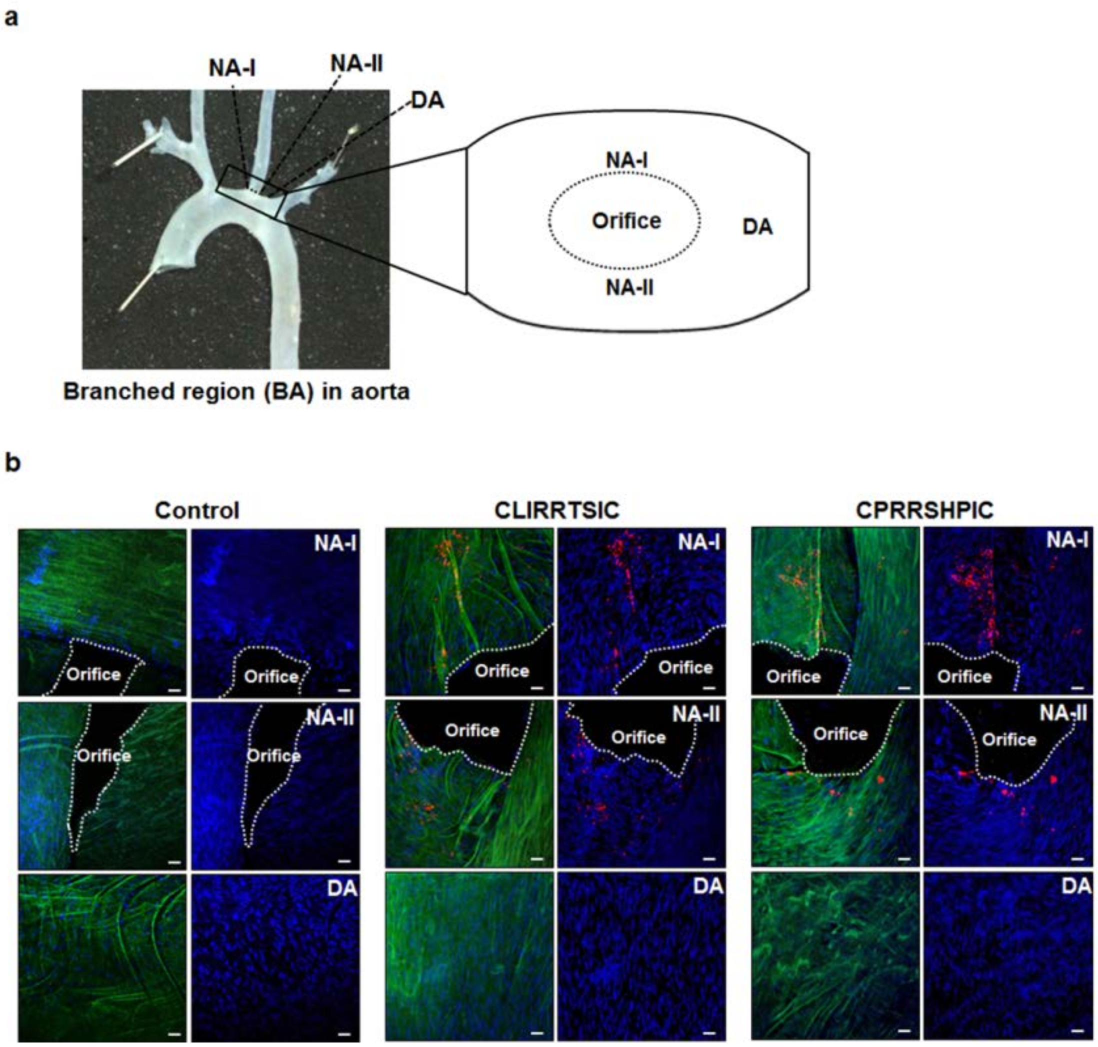

Supplementary Figure 3. *In vivo* binding of selected peptides (SPs)-displaying phages unto endothelium of branched regions of aorta (BA) in mice.

The control phages, CLIRRTSIC- or CPRRSHPIC-displaying phages (2×10^{11} pfu) were injected intravenously into C57BL/6 mice (n=3) at 3 days post-LCA partial ligation, and allowed to circulate for 3 h. (a) Appearance and anatomical diagram of branched region in mouse aorta. (b) The aorta was removed and the level of phage binding evaluated by *en face* staining. Aortic tissues were fixed and bound phages were stained using an anti-M13 bacteriophage antibody (red); nuclei were stained with DAPI (blue). Phage binding was compared in both near areas of orifice of branch vessels

(NA-I and NA-II) with disturbed flow and distant area (DA) using confocal microscopy (red: phage, blue: nuclei, green: elastic lamina of vessel) (magnification, ×400; scale bars, 10 μm).

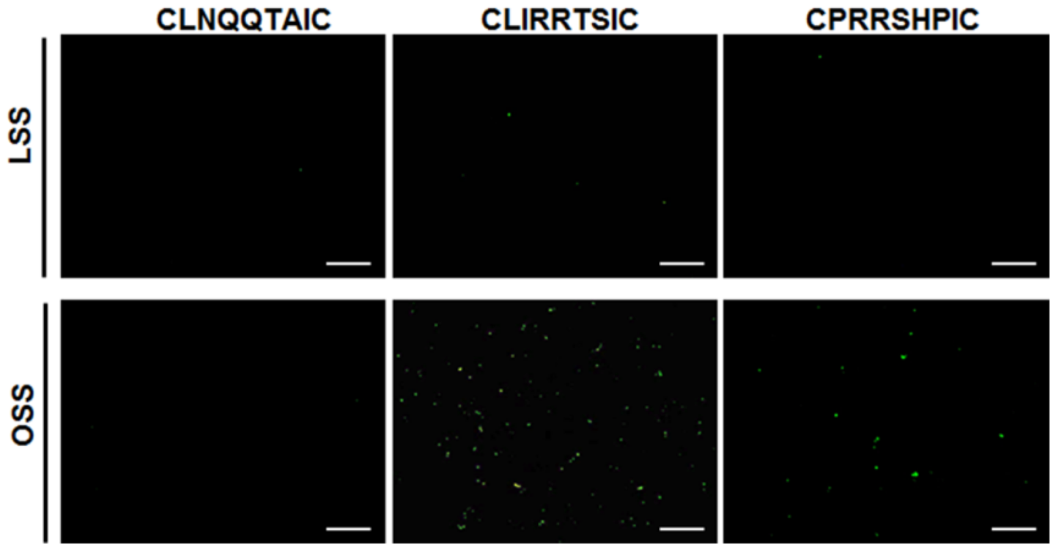

Supplementary Figure 4. Specificity of synthetic SPs to pro-atherogenic ECs.

HUVECs were exposed to flow or static conditions for 48 h. Cells were incubated with biotin-labeled control CLNQQTAIC, CLIRRTSIC, or CPRRSHPIC peptide (20 μM) for 4 h. Attached peptides were stained with streptavidin conjugated Qdot (green) (magnification, ×100; scale bars, 100 μm).

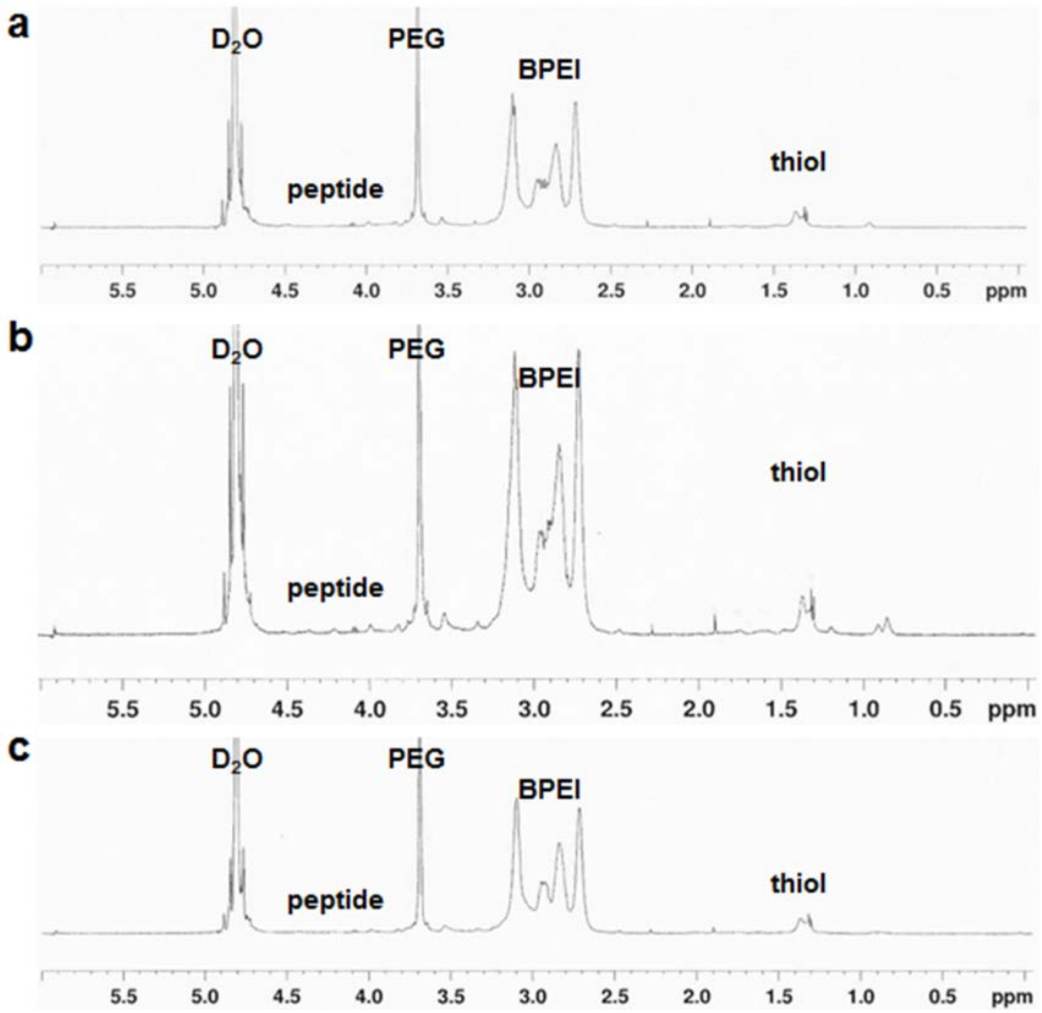

Supplementary Figure 5. ^{1}H NMR spectra of BPEI-SS-PEGCLNQQTAIC (a), BPEI-SS-PEG-CLIRRTSIC (b) and BPEI-SS-PEG-CPRRSHPIC (c) .

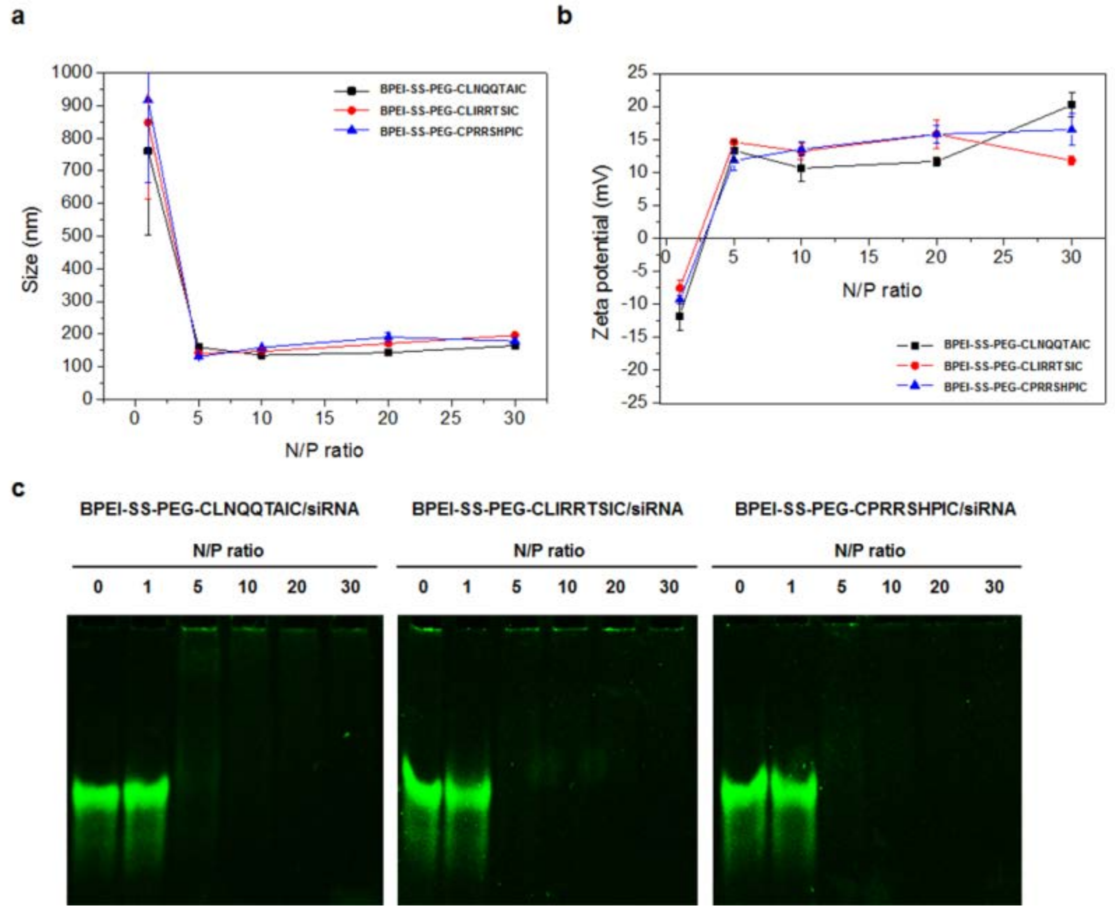

Supplementary Figure 6. Physicochemical properties of the polyplexes.

The polyplexes were prepared at a various N/P ratio by adding the peptides-conjugated polymer solution to the siRNA solution and incubated for 30 min at room temperature. (a) Size and (b) zeta potential measurements, and (c) agarose gel retardation assays of polyplexes of BPEI-SS-PEG-CLNQQTAIC, BPEI-SS-PEG-CLIRRTSIC and BPEI-SS-PEG-CPRRSHPIC. Data represent mean ± SEM (n=3).

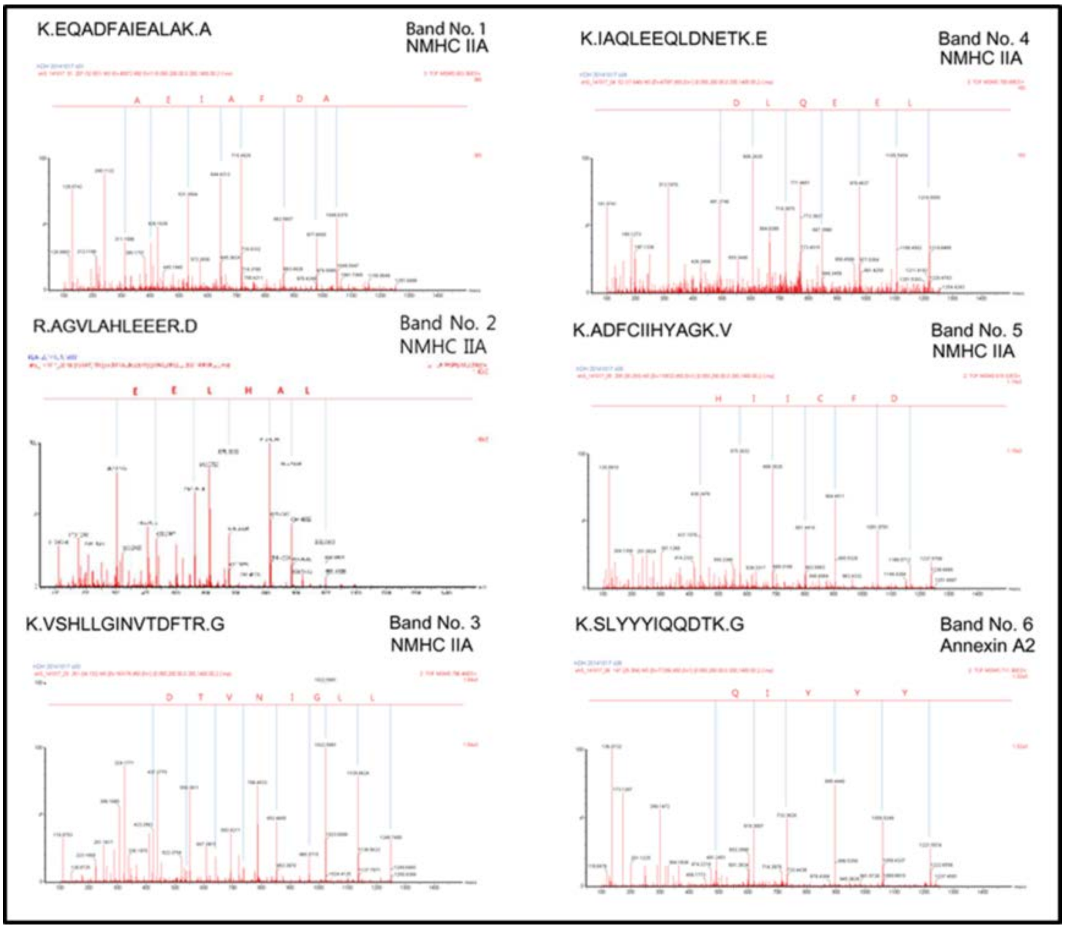

Supplementary Figure 7. Representative MS/MS spectra of CLIRRTSIC peptide-binding proteins.

Confluent HUVECs were exposed to LSS or OSS for 48 h and incubated with biotinylated CLIRRTSIC peptide for 4 h. Cell lysates were precipitated using streptavidin beads to pull down the biotinylated CLIRRTSIC peptide-binding proteins and were resolved by SDS-PAGE. Six proteins extracted from the silver stained gels were sequenced by liquid chromatography-tandem mass spectrometry. MS/MS spectra of $[M+H]^+$ ions of one of the peptides derived from the indicated protein is shown.
